# Impact of Vaccination on Intra-Host Genetic Diversity of Patients Infected with SARS-CoV-2 Gamma Lineage

**DOI:** 10.3390/v16101524

**Published:** 2024-09-26

**Authors:** Beatriz de Carvalho Marques, Cecília Artico Banho, Lívia Sacchetto, Andreia Negri, Nikos Vasilakis, Maurício Lacerda Nogueira

**Affiliations:** 1 Laboratório de Pesquisas em Virologia, Faculdade de Medicina de São José do Rio Preto, São José do Rio Preto 15090-000, São Paulo, Brazil; bbiacarvalhomarques@gmail.com (B.d.C.M.); ceciabanho@gmail.com (C.A.B.); liviasacchetto@gmail.com (L.S.); 2 Vigilância Epidemiológica, Secretaria de Saúde de São José do Rio Preto, São José do Rio Preto 15090-000, São Paulo, Brazil; andreiafrancesli@yahoo.com.br; 3 Department of Pathology, University of Texas Medical Branch, Galveston, TX 77555, USA; nivasila@utmb.edu; 4 Center for Vector-Borne and Zoonotic Diseases, University of Texas Medical Branch, Galveston, TX 77555, USA; 5 Institute for Human Infection and Immunity, University of Texas Medical Branch, Galveston, TX 77555, USA

**Keywords:** COVID-19, vaccination, Gamma lineage, negative selection, breakthrough infections

## Abstract

The high transmissibility, rapid evolution, and immune escape of SARS-CoV-2 variants can influence the course of infection and, in turn, morbidity and mortality in COVID-19, posing a challenge in controlling transmission rates and contributing to the emergence and spread of new variants. Understanding the factors that shape viral genetic variation is essential for comprehending the evolution and transmission of SARS-CoV-2, especially in vaccinated individuals where immune response plays a role in the progression and spread of this disease. In this context, we evaluated the impact of immunity induced by the CoronaVac vaccine (Butantan/Sinovac) on intra-host genetic diversity, analyzing 118 whole-genome sequences of SARS-CoV-2 from unvaccinated and vaccinated patients infected with the Gamma variant. Vaccination with CoronaVac favors negative selection at the intra-host level in different genomic regions. It prevents greater genetic diversity of SARS-CoV-2, reinforcing the importance of vaccination in reducing the emergence of new mutations and virus transmission.

## 1. Introduction

Variants of the severe acute respiratory syndrome coronavirus 2 (SARS-CoV-2) emerged due to rapid virus evolution as well as high transmission rates, especially in Variants of Concern (VOCs) such as Alpha (B.1.1.7) [1], Beta (B.1.351) [2], Gamma [3], Delta (B.1.617.2) [4], and Omicron (B.1.1.529) [5]. These variants are characterized by specific mutations along the ~30 kilobase (kb) genome composed of 14 open reading frames (ORFs), as follows: ORF1a and ORF1b, which produce the non-structural proteins (Nsps) Nsp1 to Nsp16; four structural proteins (spike [S], membrane [M], envelope [E], and nucleocapsid [N]); and some accessory proteins, including ORF3a, ORF6, ORF7a, ORF7b, ORF8, ORF9, and ORF10 [6].

The COVID-19 pandemic spurred rapid vaccine development, as herd immunity can prevent severe disease in different age groups [7,8]. In Brazil, CoronaVac (Butantan Institute/Sinovac Biotech), an inactivated whole-virus vaccine, was first approved for emergency use in January 2021 [9]; its wide distribution helped to decrease severe cases and deaths [9]. However, the rapid evolution of SARS-CoV-2, which resulted in a high occurrence of mutations that allied to the emergence of new VOCs after the distribution of vaccines in Brazil, made controlling viral transmission a challenge due to the escape of natural or vaccine-acquired immunity [10,11].

Although the diversity of SARS-CoV-2 in viral populations has been demonstrated [12,13,14,15], little information is available on how specific vaccine types may exert selective pressures and shape virus evolution [16]. To better understand the intra-host diversity of SARS-CoV-2 after vaccination in Brazil in early 2021, we investigated 118 whole viral genomes from unvaccinated and vaccinated patients who were diagnosed with COVID-19 from April to July 2021 in São José do Rio Preto and surrounding cities in the state of São Paulo, Brazil. Patients were divided into two equal groups, as follows: (1) unvaccinated individuals (infected with SARS-CoV-2 who did not receive any dose of vaccine); and (2) vaccinated individuals (infected with SARS-CoV-2 14 or more days after having received their second dose of the CoronaVac vaccine—breakthrough infections). We found that the CoronaVac vaccine favored negative selection in different regions of the SARS-CoV-2 genomes, thus reducing genetic diversity at the intra-host level. This outcome reinforces the importance of vaccination as a tool to prevent the emergence of new variants that may enhance virus fitness and affect the progression of COVID-19 worldwide.

## 2. Materials and Methods

### 2.1. Samples

Nasopharyngeal swab samples from COVID-19 cases were collected at the Hospital de Base de São José do Rio Preto (HB), a medical center serving over 2 million residents across 102 municipalities that is part of the 15th Regional Health Division (RHD XV) of the state of São Paulo. The samples were sent to the Laboratório de Pesquisas em Virologia (LPV) at the Faculdade de Medicina de São José do Rio Preto (FAMERP), where they were identified, aliquoted, and stored in a freezer at −80 °C until RT-qPCR was performed to confirm SARS-CoV-2 positivity and for subsequent analyses. These samples were obtained from São José do Rio Preto residents and residents from surrounding cities diagnosed with COVID-19 between 14 April and 15 July 2021.

Based on the immunization criteria, 118 samples were selected and divided into two groups, as follows: (1) 59 samples from unvaccinated patients with ages ranging from two to 65 years (average age: 31 years); and (2) 59 samples from fully immunized patients (individuals who received two doses of CoronaVac (Butantan Institute/Sinovac Biotech) who were diagnosed with COVID-19 more than 14 days after having completed the two-dose vaccination schedule), with ages ranging from 24 to 91 years (average: 60 years). Our sample groups were selected based on the vaccination schedule in the state of São Paulo, which, prior to the time of sampling, included men and women over 37 years of age, pregnant women, Indigenous people, Quilombolas (Afro-Brazilian descendants of the escaped enslaved living in settlement communities), immunocompromised individuals, patients with comorbidities, health professionals, public security and prison administration workers, public transport staff, and education professionals.

### 2.2. Ethics

This study was approved by the institutional review board of the Faculdade de Medicina de São José do Rio Preto (protocol: 31588920.0.0000.5415, approved on 29 November 2021). Informed consent was not required since all samples were collected for routine diagnosis and the data were analyzed anonymously, ensuring total confidentiality for all participants.

### 2.3. Molecular Investigation

Total RNA was extracted from 140 µL of the nasopharyngeal swab samples using a QIAamp Viral RNA Mini Kit (QIAGEN, Hilden, Germany), following the manufacturer’s instructions. SARS-CoV-2 RNA was investigated with a one-step, real-time polymerase chain reaction (RT-qPCR) using primers and probes targeting the envelope (E), the nucleocapsid (N), and human RNAse P, using the GeneFinder COVID-19 Plus RealAmp Kit (OSANG Healthcare, KOR) [17]; RT-qPCR was conducted in a QuantStudio 3 Real-Time PCR System (Thermo Fisher Scientific, Waltham, MA, USA), according to the manufacturer’s instructions. The results were interpreted based on the cycle quantification value (Cq), in which samples presenting Cq less or equal to 40 were considered positive, as recommended in the kit instructions. Positive and negative controls included in the GeneFinder COVID-19 Plus RealAmp Kit (non-infectious DNA plasmids coding for the SARS-CoV-2 E gene and N gene) were used in the assay.

### 2.4. Whole-Genome Sequencing

Whole-genome sequencing was performed through cDNA synthesis, whole-genome amplification, and through library preparation following the instructions provided with the Illumina CovidSeq Test (Illumina, USA). The quality and size of the libraries were verified using the Agilent 4150 TapeStation system (Agilent, Santa Clara, CA, USA). The libraries were pooled in equimolar concentrations, and sequencing was conducted using an Illumina MiSeq system with a MiSeq Reagent Kit v2 (2 × 150 cycles) (Illumina, San Diego, CA, USA).

### 2.5. Genome Assembling and Variant Analyses

The quality of raw reads was checked using the FastQC v.0.11.4 analysis tool [18]. Cutadapt v.4.6 [19] was used to filter out low-quality reads, low-quality bases, reads with a minimal length of 75 base pairs (bp), and primer removal. The cleaned, paired-end reads were mapped against the Wuhan-Hu-1 reference genome (NC_045512.2) using BWA-mem 0.7.17 software [20]. Post-processing steps, PCR duplicate removal, and a consensus sequence were generated using SAMtools v1.10 [20,21] and iVar [22]. The genomes were submitted to the Pangolin COVID-19 Lineage Assigner Tool version v.4.0.5 to confirm the variant classification [23].

After lineage identification, only sequences classified as Gamma lineage were selected for the subsequent analyses to avoid mutation bias from different SARS-CoV-2 variants. Intra-host single nucleotide variant (iSNV) analysis was conducted using LoFreq v.2.1.5 [24]. We identified iSNVs with the following criteria: minimum coverage of 100 mapped reads for each genome position; base quality >30; and minor alternative allele frequency (AAF) ≥ 5%. The biological effects of the identified iSNVs were annotated using SnpEff v.2.0.5, with default settings [25]. Plots to visualize the AAFs for all samples were generated in R v.3.6.1 software [26], employing the ggplot2 package [27].

### 2.6. Evolutionary Analyses

The inference of selective pressures on a particular region/gene can be manifested as diversifying or purifying selection. We used a combination of two evolutionary analyses to enhance the detection of relevant sites in the SARS-CoV-2 genome in unvaccinated and vaccinated individuals. Fixed Effects Likelihood (FEL) was used to identify sites experiencing pervasive diversifying or purifying selection [28], and the Mixed Effects Model of Evolution (MEME) was used to detect sites undergoing both pervasive and episodic diversifying selection. Both methods were implemented in HyPhy v.2.5.32 software [29] and used a Maximum Likelihood to infer non-synonymous (dN) and synonymous (dS) substitution rates on a per-site basis for the given coding alignment and the corresponding phylogenetic tree [29].

### 2.7. Statistics

The collected data were entered into an Excel spreadsheet (Microsoft, Redmond, WA, USA) and imported into R [26] for statistical analyses. Pearson’s chi-squared test was used to compare categorical variables and determine whether the expected frequency in the groups was met. A significance level of 0.05 (5%) was adopted for all statistical tests.

## 3. Results

### 3.1. Distribution of iSNVs across SARS-CoV-2 Genomes

**Figure 1:**
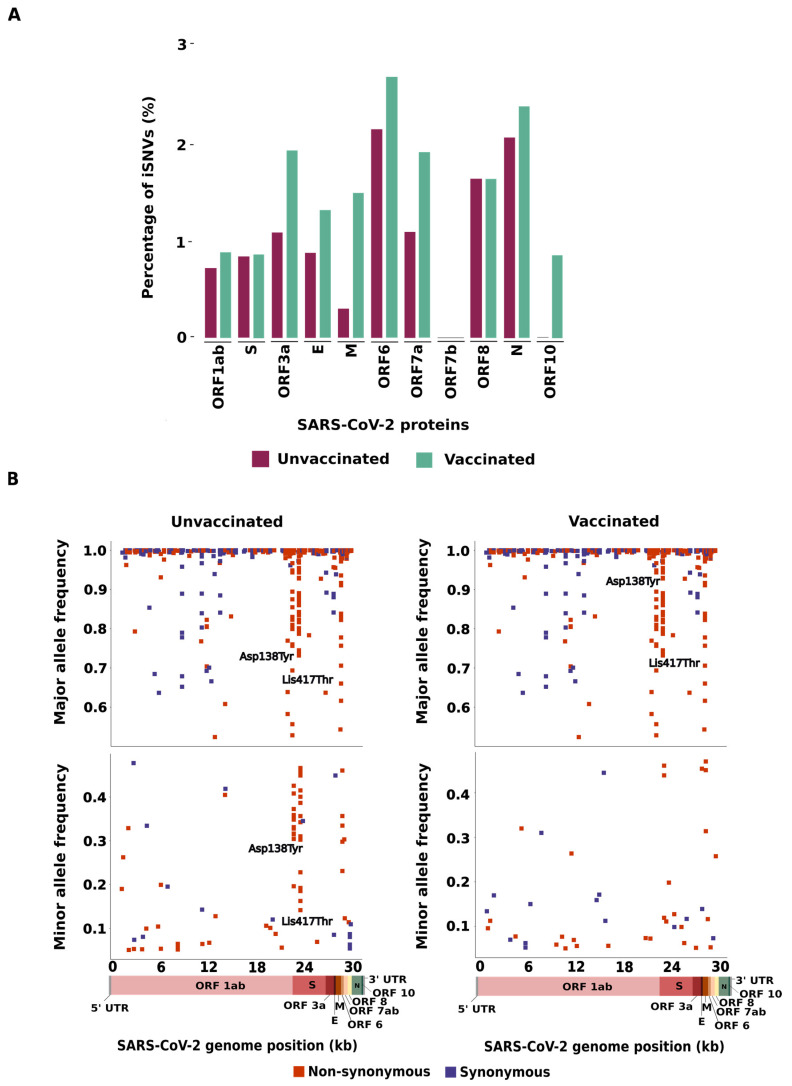
Intra-host genetic diversity of SARS-COV-2 after two doses of CoronaVac: (**A**) Percentage of iSNVs across the SARS-CoV-2 genome from unvaccinated and vaccinated patients in relation to gene size; (**B**) Major and minor allele frequency for non-synonymous (blue) and synonymous (orange) variants in the unvaccinated and vaccinated groups. Coding regions of the SARS-CoV-2 genome, based on the reference genome (NC_045512.2), are shown at the bottom of the figure. ORF: open reading frame. S: spike. E: envelope. M: membrane. N: nucleocapsid.

Analysis of within-host genetic diversity of SARS-CoV-2 genomes identified 238 and 299 iSNVs in the unvaccinated and vaccinated groups, respectively (Table 1). The distribution of iSNVs across the SARS-CoV-2 genome was identified and normalized by gene size (Figure 1A, Tables S1 and S2). For both groups, the highest percentage of iSNVs was identified in the ORF6 gene (2.15% in the unvaccinated group and 2.69% in the vaccinated group), followed by the N gene (2.06% in the unvaccinated and 2.38% in the vaccinated group). No significant difference was identified between the groups (*p* > 0.05). In general, the majority of gene-coding structural proteins (E, M, and N) showed a higher percentage of iSNVs in the vaccinated group than in the unvaccinated group, except for the S gene, which exhibited similar percentages of iSNVs in both groups. Likewise, ORF1ab, ORF3a, ORF7a, and ORF10 showed a higher percentage of iSNVs in the vaccinated group than in the unvaccinated group. The opposite was detected in ORF8. No statistical difference was identified in the non-structural proteins (*p* > 0.05).

### 3.2. Characterization of the iSNVs Detected in the SARS-CoV-2 Genomes

Next, we investigated whether the number of non-synonymous and synonymous iSNVs can be affected by the allele frequency, classifying all the detected iSNVs as major (iSNVs showing more than 50% of mapping reads to an alternative allele) or minor variants (iSNVs displaying frequency from 5–49% of mapping reads to an alternative allele). In the unvaccinated group, we found that 204/243 (83.9%) iSNVs were considered major variants, with 112/204 (54.9%) classified as non-synonymous and 92/204 (45.1%) classified as synonymous. In this same group, 39/243 (16.1%) iSNVs were classified as minor (26/39, 66.7% non-synonymous, and 13/39, 33.3% synonymous). In the vaccinated group, 269/309 (87.1%) iSNVs were considered major (151/269, 56.1% non-synonymous and 118/269, 43.9% synonymous) and 40/309 (12.9%) were considered minor (26/40, 65% non-synonymous and 14/40, 35% synonymous) (Figure 1B, Tables S3 and S4).

We also analyzed the number of shared and exclusive iSNVs found in SARS-CoV-2 genomes from the vaccinated and unvaccinated groups (Tables S5 and S6). A total of 84 shared iSNVs were identified, 70 of which (83.3%) did not correspond to Gamma lineage-defining mutations. Most of the shared iSNVs were distributed through ORF1ab (44/84, 52.39%) and S (16/84, 19.05%) (Table S5). Moreover, it is worth noting that some Gamma lineage-defining mutations were lost in both groups, as follows: the amino acid substitutions S:Glu484Lys (lost in 20 sequences from both the unvaccinated and vaccinated groups); S:Asn501Tyr (absent in 19 and 20 sequences in the unvaccinated and vaccinated groups, respectively); S:His655Tyr (lost in 27 sequences in both groups); and ORF3a:Gly174Cys, which was lost in all the genome sequences analyzed (Table S5). We found 154 iSNVs exclusive to the unvaccinated group and 215 iSNVs exclusive to the vaccinated group; most of these were classified as non-synonymous mutations (Table S6). The number of shared or exclusive iSNVs identified in a single patient sample were also analyzed; we found a higher prevalence of iSNVs in only one sequence in the vaccinated group (*n* = 221/299, 73.9%) compared with the unvaccinated group (*n* = 161/238, 67.6%) (Tables S1 and S2).

### 3.3. Allele Composition of the SARS-CoV-2 Genomes

Analysis of the allele composition of each site showed that the most prevalent substitution was C>T in both groups (unvaccinated: *n* = 118/238, 49.6%; vaccinated: *n* = 141/299, 47.2%), for structural (24/118, 20.3% in the unvaccinated and 32/141, 22.7% in the vaccinated group) and non-structural proteins (94/118, 79.7% in the unvaccinated and 109/141, 77.3% in the vaccinated group) (Tables S1 and S2). However, by normalizing the number of nucleotide substitutions by gene size, we found a higher frequency of this transition in ORF6 than in other genomic regions in the unvaccinated group. In contrast, a higher density of C>T was displayed in ORF3a in the vaccinated group. After ORF6, the S gene presented the second highest number of C>T substitutions. The second most common substitution observed was G>T and G>A in sequences from unvaccinated (*n* = 35/118, 29.7%, *n* = 28/118, 23.7%, respectively) and vaccinated (*n* = 44/141, 31.2%, *n* = 27/141, 19.1%, respectively) patients.

Interestingly, we detected two genomic sites for the non-synonymous mutations with major allele frequency differences within both groups. The non-synonymous iSNV in the S region at position 21,974 corresponded to a transversion (G>T) that represents an amino acid substitution (Asp138Tyr) observed in 23 unvaccinated and 39 vaccinated patients. This mutation displayed an allele frequency ranging from 19% to 100% and 52% to 100% in SARS-CoV-2 sequences from the unvaccinated and vaccinated groups, respectively. Similarly, we identified a mutation at the 22,812 genomic position that corresponded to a transversion (A>C); this nucleotide substitution represents an amino acid change (Lys417Thr) found in 43 unvaccinated and 56 vaccinated patients, with an allele frequency ranging from 14% to 100% and from 12% to 100% in the unvaccinated and vaccinated groups, respectively (Figure 1B).

### 3.4. Selection Pressures Detected in the SARS-CoV-2 Genomes

Finally, to better understand the selective pressures that shape the intra-host evolution of SARS-CoV-2 in unvaccinated and vaccinated individuals, we used a combination of two tests to detect selection signatures across the SARS-CoV-2 genome. Our analyses identified ten sites in five proteins (ORF1ab, S, M, ORF6, and ORF7a) under negative selection and one in ORF1ab under positive selection in unvaccinated patients (Table 2). In contrast, in vaccinated individuals, we identified 26 sites under negative selection distributed across seven proteins (ORF1ab, ORF3a, E, ORF6, ORF7a, ORF8, and N), and three sites under positive selection located in ORF1ab and N (Table 2).

**Figure 2:**
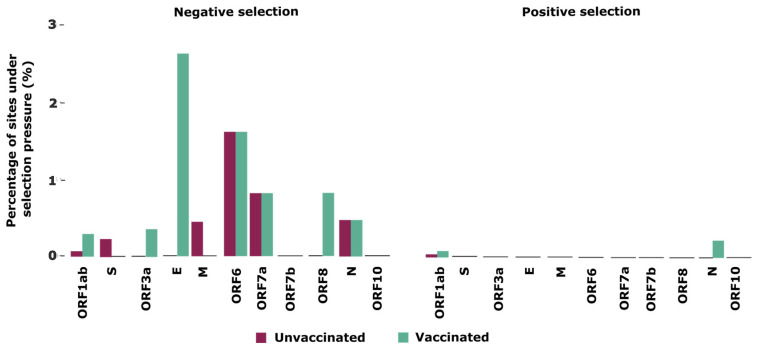
Comparison of selective pressure on SARS-CoV-2 genome sites between unvaccinated and CoronaVac-vaccinated patients infected with the Gamma lineage. ORF: open reading frame. S: spike. E: envelope. N: nucleocapsid. M: membrane.

When normalizing the number of sites under selection by gene size, we noticed that E showed the highest percentage (2.63%) of sites under negative selection in the vaccinated group compared to other genomic regions and the unvaccinated group. The second protein with the highest proportion of sites under negative selection was ORF6 (1.61%) for both groups (Figure 2). For sites under positive selection, N and ORF1ab were the only proteins found in both the unvaccinated and vaccinated groups.

## 4. Discussion

Vaccines are the primary measure used to reduce the transmission of SARS-CoV-2 and mitigate severe COVID-19 cases and related deaths [30]; however, some studies have suggested that vaccination against the virus could increase intra-host selection pressures for immune-escape mutations [31,32]. Our study of unvaccinated and vaccinated individuals infected with the SARS-CoV-2 Gamma lineage showed different results. We found similar average numbers of iSNVs in samples from vaccinated and unvaccinated patients, corroborating previous studies [16,33] and confirming that vaccination with CoronaVac does not favor intra-host genetic diversity in patients with breakthrough infections. It is important to note that this result may not be the same for all SARS-CoV-2 lineages or vaccines; for example, in 2023, Gu et al. reported that the incidence of iSNVs in patients infected with the Delta lineage who had received two doses of the Comirnaty (BNT162b2) vaccine was significantly higher than in unvaccinated patients [16]. Similarly, according to Jena et al., Delta and Omicron lineages showed a higher occurrence of iSNVs after vaccination with Covaxin (Bharat Biotech) and Covishield (AstraZeneca), which could be related to the increase in immune escape variations in late 2021, when these variants showed a peak of infections [34].

It is crucial to consider various factors when evaluating iSNVs, such as their allele frequency and impact on changing protein or gene function, in order to fully comprehend virus evolution. We evaluated the number of iSNVs detected in only one sample in both groups. Interestingly, we observed that although a greater number of iSNVs were distributed across the SARS-CoV-2 genome of the vaccinated group, most of these iSNVs (73.9%) ranged from 5% to 49% in allele frequency, meaning that these mutations are sporadic and may not be fixed in the viral population. Similarly, Gu et al. showed that over 70% of the iSNV sites identified in their samples were uniquely observed in a single patient [16]. Our findings reinforce that minor iSNVs do not provide relevant information for understanding the diversity of SARS-CoV-2 in vaccinated and unvaccinated individuals. In this way, we demonstrate that vaccination with the CoronaVac vaccine does not enhance the mutation rate or change the mutation profile of SARS-CoV-2 Gamma lineage variants.

We detected several iSNVs with allele frequencies greater than 50%, including many Gamma-defining mutations. Among these, two non-synonymous mutations, S: D138Y and S: K417T, showed highly variable allele frequencies in samples from both groups. These two critical locations involve amino acid substitutions that can impact binding by monoclonal and polyclonal antibodies, influencing host cell entry and, in turn, transmissibility [3]. While we found iSNVs displaying different allele frequencies throughout the entire genome of SARS-CoV-2 from vaccinated and unvaccinated individuals, a wide difference in allele frequency was identified in the S gene, reinforcing its mutational potential, which has already been mentioned in other studies [30,35,36].

Further analyses of iSNVs determined that the unvaccinated and vaccinated groups displayed similar percentages of non-synonymous and synonymous mutation, and we found no association between mutation class and vaccination status. These results are corroborated by previous studies showing that non-synonymous mutations were the most frequent alteration in SARS-CoV-2 samples worldwide [37]. Even so, synonymous mutations were detected in over 40% of all nucleotide substitutions and still require careful examination since they can affect codon usage, maintenance of the secondary RNA structure, and long-term translation efficiency [38]. Furthermore, we did not identify any significant genetic variation in any protein (including the S); this ran counter to other studies, which showed that variations in the amino acid sequences of this protein could influence interaction with the host receptor, pathogenesis, viral replication, infectivity, and transmissibility [35,39]. We also analyzed the number of iSNVs normalized by protein size. We found no significant differences in the genetic diversity of structural and non-structural proteins in unvaccinated and vaccinated patients, suggesting that CoronaVac does not favor the intra-host diversity of SARS-CoV-2 in any genomic hotspot, which was previously verified by other studies using different vaccine technologies [16].

Additionally, we showed that C>T substitution was the most frequent SNP detected. This is in line with previous findings demonstrating that the C>T transition was responsible for 55.1% of all SARS-CoV-2 mutations identified in 2020; the G>T transversion G>T (found in this study as the second most common in the S gene) was the most common nucleotide substitution in the SARS-CoV-2 genome worldwide [33,38]. The C>T mutational event has been implicated as important for controlling virus replication since the excessive occurrence of this transition is linked to a host APOBEC-like (apolipoprotein B mRNA editing) process that plays a role in antiviral defense against retroviruses and may drive several mutational hot spots in the SARS-CoV-2 genome without providing an adaptive advantage to the virus but still affecting its rate of evolution [38,40].

We also analyzed the selective pressures at genomic sites to better understand how vaccination with CoronaVac may influence genetic diversity in the SARS-CoV-2 genomes. In general, we identified a greater occurrence of negative selection throughout the SARS-CoV-2 genome, especially in the vaccinated group, showing that CoronaVac can modulate the evolution of SARS-CoV-2 over a short time. Similar results have already been found for other RNA viruses [41] such as influenza [42], dengue [43], chikungunya [44], and SARS-CoV-2 [13,42]. Here, however, we demonstrated that negative selection was favored in vaccinated individuals when this vaccine was the most widely distributed in Brazil in 2021, especially in E and ORF6, proteins responsible for suppressing the immune response of cells infected by the SARS-CoV-2 virus, modulating the inflammatory response and potentially helping the virus to evade detection by the immune system, acting as an important target of selection [45,46]. CoronaVac has been implicated in producing T cells specific to several SARS-CoV-2 antigens [47], which may confer an advantage in clearing virus infection [48]. In this way, CoronaVac controls transmission and pathogenesis through humoral and cellular immune responses against different virus proteins, and negative selection pressure against low-frequency variants on most viral proteins, suggesting its role in reducing virus diversity.

Although our findings demonstrate CoronaVac’s influence in preventing the intra-host diversity of SARS-CoV-2, it is important to note that this study has limitations. To minimize potential bias caused by mutations of different variants of SARS-CoV-2, we did not compare vaccinated and unvaccinated patients presenting breakthrough infections with other lineages. Similarly, we could not assess whether the selective pressures verified in individuals who received CoronaVac would be the same if other vaccine technologies were used, because, during the sampling period, the Gamma lineage was the dominant circulating VOC (reaching more than 90%) [9]. Furthermore, CoronaVac was the first vaccine to be licensed and widely used in the country during this same period. Since then, several lineages have been introduced [49], and other licensed SARS-CoV-2 vaccines have been administered, complicating this kind of study. Moreover, it is important to emphasize that, because we opted to analyze sequences from patients who did not receive any vaccine against SARS-CoV-2, our sampling time was limited to three months, a short period for observing the effects of selective pressures on the virus genome.

Our data, together with neutralization studies of other vaccine technologies and other SARS-CoV-2 lineages, encourage the continued administration of vaccines against COVID-19. Trombetta et al. [50] showed that antibodies generated after triple-mRNA vaccination (mRNA 1273, Moderna, and/or Comirnaty, BNT162b2), or after natural SARS-CoV-2 infection, combined with a two-dose vaccine result in the highest neutralizing capacity against the Omicron BA.1 variant [50]. Similarly, Girl et al. [51] showed different levels of neutralization of the Alpha, Beta, Delta, and Omicron lineages after vaccination with Vaxzevria (AstraZeneca), Comirnaty (Pfizer), and Spikevax (Moderna). However, all were higher than the wild-type virus [51]. Such differences in neutralization levels indicate the need for continued study of updated COVID-19 vaccines to maintain disease control.

Although SARS-CoV-2 vaccine efficacy decreases over time [52], especially against rapidly evolving viruses, and despite the demonstrated ability of SARS-CoV-2 lineages to escape neutralizing antibodies [53,54], our findings highlight that two doses of CoronaVac vaccine favor negative selection in the structural and non-structural genes of SARS-CoV-2 obtained from patients infected with the Gamma lineage. This study suggests that vaccination is important to reduce the emergence of new variants at the intra-host level, preventing SARS-CoV-2 genetic diversity and the emergence of new and concerning mutations that may confer higher adaptative value to SARS-CoV-2 variants.

## Figures and Tables

**Table 1:** Number of non-synonymous (NS) and synonymous (S) iSNVs found in the SARS-CoV-2 genome from patients unvaccinated and vaccinated with CoronaVac.

	Unvaccinated		Vaccinated	
**Region**	**NS (%)**	**S (%)**	**NS (%)**	**S (%)**
**ORF1ab**	79 (58.5)	74 (71.8)	92 (54.8)	96 (73.3)
**S**	20 (14.8%)	12 (11.7)	27 (16.1)	6 (4.6)
**ORF3a**	9 (6.7)	0 (0.0)	13 (7.7)	3 (2.3)
**E**	0 (0.0)	2 (1.9)	0 (0.0)	3 (2.3)
**M**	0 (0.0)	2 (1.9)	3 (1.8)	7 (5.3)
**ORF6**	3 (2.2)	1 (1.0)	3 (1.8)	2 (1.5)
**ORF7a**	2 (1.5)	2 (1.9)	4 (2.4)	3 (2.3)
**ORF7b**	0 (0.0)	0 (0.0)	0 (0.0)	0 (0.0)
**ORF8**	6 (4.4)	0 (0.0)	5 (3.0)	1 (0.8)
**N**	16 (11.9)	10 (9.8)	20 (11.9)	10 (7.6)
**ORF10**	0 (0.0)	0 (0.0)	1 (0.5)	0 (0.0)
**TOTAL**	135 (100)	103 (100)	168 (100)	131 (100)

**Table 2:** Sites under positive or negative selection for each SARS-CoV-2 coding region analyzed using MEME and FEL.

	Unvaccinated		Vaccinated	
Locus	Positive Selection	Negative Selection	Positive Selection	Negative Selection
**ORF1ab**	NSP6 (106)	NSP3 (106, 681), NSP10 (82), NSP13 (495)	NSP3 (1303), NSP6 (107)	NSP2 (91,443), NSP3 (236, 394, 447, 662, 1092, 1121, 1742), NSP6 (76, 138), NSP10 (16), NSP13 (237, 356), NSP14 (302, 373), NSP15 (278), NSP16 (178)
**S**	0	554, 995, 1065	0	0
**ORF3a**	0	0	0	43
**E**	0	0	0	8, 23
**M**	0	53	0	0
**ORF6**	0	49	0	61
**ORF7a**	0	88	0	11
**ORF8**	0	0	0	75
**N**	0	0	200	194, 363
**ORF10**	0	0	0	0

ORF: open reading frame. S: spike. E: envelope. N: nucleocapsid. M: membrane.

## Data Availability

All data generated or analyzed during this study are available in the Mendeley Data repository, DOI: 10.17632/rcxddvxv3c.2.

## Supplementary Materials

The following supporting information can be downloaded at: https://www.mdpi.com/article/10.3390/v16101524/s1. All data used to perform the analyses and graphs are available in Table 1, Table 2, and Supplementary Tables S1–S7. All SARS-CoV-2 genomes generated and analyzed in this study are available in the GenBank database (https://www.ncbi.nlm.nih.gov/genbank/), and their respective access numbers are provided in Supplementary Table S7.
