# Supplementary material for: Impact of Vaccination on Intra-Host Genetic Diversity of Patients Infected with SARS-CoV-2 Gamma Lineage
Source: Viruses. 2024 Sep 26;16(10):1524. doi: 10.3390/v16101524 (PMC11512383; doi:10.3390/v16101524)
Supplement: Supplementary file 1 [file viruses-16-01524-s001.zip › viruses-3175978 -Table S1.pdf]

Table S1. Number of non-synonymous (NS) and synonymous (S) iSNVs found in the SARS-CoV-2 genome from patients unvaccinated and vaccinated with CoronaVac.

	**Unvaccinated**		**Vaccinated**	
**Region**	**NS (%)**	**S (%)**	**NS (%)**	**S (%)**
**ORF1ab**	79 (58.5)	74 (71.8)	92 (54.8)	96 (73.3)
**S**	20 (14.8%)	12 (11.7)	27 (16.1)	6 (4.6)
**ORF3a**	9 (6.7)	0 (0.0)	13 (7.7)	3 (2.3)
**E**	0 (0.0)	2 (1.9)	0 (0.0)	3 (2.3)
**M**	0 (0.0)	2 (1.9)	3 (1.8)	7 (5.3)
**ORF6**	3 (2.2)	1 (1.0)	3 (1.8)	2 (1.5)
**ORF7a**	2 (1.5)	2 (1.9)	4 (2.4)	3 (2.3)
**ORF7b**	0 (0.0)	0 (0.0)	0 (0.0)	0 (0.0)
**ORF8**	6 (4.4)	0 (0.0)	5 (3.0)	1 (0.8)
**N**	16 (11.9)	10 (9.8)	20 (11.9)	10 (7.6)
**ORF10**	0 (0.0)	0 (0.0)	1 (0.5)	0 (0.0)
**TOTAL**	135 (100)	103 (100)	168 (100)	131 (100)
