# Supplementary material for: Impact of Vaccination on Intra-Host Genetic Diversity of Patients Infected with SARS-CoV-2 Gamma Lineage
Source: Viruses. 2024 Sep 26;16(10):1524. doi: 10.3390/v16101524 (PMC11512383; doi:10.3390/v16101524)
Supplement: Supplementary file 1 [file viruses-16-01524-s001.zip › viruses-3175978 -Table S2.pdf]

Table S2. Sites under positive or negative selection for each SARS-CoV-2 coding region analyzed using MEME and FEL.

	Unvaccinated		Vaccinated	
**Locus**	**Positive Selection**	**Negative Selection**	**Positive Selection**	**Negative Selection**
**ORF1ab**	NSP6 (106)	NSP3 (106, 681), NSP10 (82), NSP13 (495)	NSP3 (1303), NSP6 (107)	NSP2 (91,443), NSP3 (236, 394, 447, 662, 1092, 1121, 1742), NSP6 (76, 138), NSP10 (16), NSP13 (237, 356), NSP14 (302, 373), NSP15 (278), NSP16 (178)
**S**	0	554, 995, 1065	0	0
**ORF3a**	0	0	0	43
**E**	0	0	0	8, 23
**M**	0	53	0	0
**ORF6**	0	49	0	61
**ORF7a**	0	88	0	11
**ORF8**	0	0	0	75
**N**	0	0	200	194, 363
**ORF10**	0	0	0	0

ORF: open reading frame. S: spike. E: envelope. N: nucleocapsid. M: membrane.
